# Hidden in Plain Sight: The Enduring Challenge of Neglected Tropical Diseases in Khartoum State, Sudan

**DOI:** 10.1155/jotm/7385292

**Published:** 2026-01-26

**Authors:** Babiker Mohamed Rahamtalla

**Affiliations:** ^1^ Department of Community Medicine, University of Medical Sciences and Technology, P.O. Box 12810 Obaid Khatim Street, Riyadh, Khartoum, Sudan, umst-edu.com

**Keywords:** epidemiology, Khartoum State, neglected tropical diseases, NTDs, prevalence, public health, schistosomiasis, soil-transmitted helminthiases, Sudan, trachoma

## Abstract

**Background:**

Neglected tropical diseases (NTDs) disproportionately affect impoverished populations in tropical regions. Despite their significant health burden, data on NTD prevalence and distribution are limited in many areas, including Khartoum State, Sudan.

**Objective:**

This study aimed to assess the prevalence and geographical distribution of NTDs in Khartoum State, Sudan, to inform targeted control interventions and elimination strategies.

**Methods:**

A cross‐sectional study was conducted using annual statistical reports from Khartoum State Ministry of Health for 2020 and 2021. Data on the prevalence of NTDs were extracted, focusing on prevalent NTDs in the state. Descriptive statistics summarized NTD prevalence. Time‐series analysis identified trends. Spatial data analysis pinpointed hotspots and clustering of NTD cases. Data consistency and accuracy were ensured.

**Results:**

Nine NTDs were prevalent: soil‐transmitted helminthiases (STHs), schistosomiasis, scabies, trachoma, snakebite envenoming, leishmaniasis, taeniasis, mycetoma, and leprosy. STH had the highest prevalence (32.7% in 2020 and 32.9% in 2021), primarily affecting children under 15. Schistosomiasis was the second most prevalent (23.0% in 2020 and 20.9% in 2021), predominantly affecting males aged 5–25. Trachoma prevalence varied across localities. Leprosy showed an increasing detection rate.

**Conclusion:**

NTDs pose a significant public health burden in Khartoum State, particularly STH and schistosomiasis. Localized control strategies, improved sanitation, hygiene, access to clean water, and integrated NTD programs are crucial to reduce the NTD burden and improve population health. Continuous surveillance is warranted, especially for leprosy.

## 1. Introduction

Neglected tropical diseases (NTDs) constitute a diverse group of parasitic and bacterial infections that disproportionately affect impoverished populations in tropical and subtropical regions. Despite their significant public health burden, NTDs have historically received limited attention in global health policy and funding priorities [1]. Although bibliometric analyses suggest a gradual increase in research output on NTDs, this remains insufficient relative to their global burden [2]. Globally, over one billion individuals are afflicted by at least one NTD, with far‐reaching implications for healthcare systems, economic productivity, and educational outcomes [3].

The epidemiological burden of NTDs is most acutely felt among youth and young adults, who have experienced a substantial share of disability‐adjusted life years (DALYs) attributed to NTDs between 1990 and 2019 [4]. Regional patterns of control and prevalence vary widely. Central Latin America, for instance, has struggled to sustain progress over 2 decades, even with substantial intervention programs [5]. In South Asia, the persistence of NTDs is driven by structural determinants such as poverty, inadequate healthcare access, and environmental factors [6]. Similarly, Southeast Asia, including Myanmar, continues to harbor multiple endemic NTDs, despite international support and health initiatives [7]. In Lebanon, socioeconomic disparities and the influx of refugees have contributed to sustained NTD prevalence [8], while Oceania faces interregional disparities in both disease burden and intervention success [9].

Recent innovations in geostatistical methods have enhanced the efficiency of prevalence survey design and execution, particularly for soil‐transmitted helminths and similar infections [10]. These tools have enabled refinement of survey protocols in low‐resource contexts [11] and encouraged the adoption of geospatial approaches in disease surveillance and resource allocation [12]. However, challenges persist, especially in postelimination surveillance. Sustainable integration of monitoring systems into national health frameworks remains elusive [13]. For instance, a global review of foodborne trematodes revealed substantial data limitations that undermine targeted control strategies [14]. Furthermore, long‐term analyses suggest that climate change, urbanization, and migration are reshaping the global landscape of NTD burden [15].

India’s multifaceted efforts against NTDs illustrate both notable achievements and ongoing gaps, offering lessons for other endemic countries [16]. In Cameroon, studies from traditional health centers underscore the continuity of local transmission, reinforcing the importance of integrating community‐based interventions [17]. Conflict‐affected countries in the Middle East and North Africa face compounding challenges in managing NTDs amid humanitarian crises and strained health systems [18]. In Brazil, research from Minas Gerais State has revealed the interplay between environmental, economic, and social determinants in shaping disease patterns [19], while macroeconomic assessments link NTDs to reduced productivity and growth across African nations [20]. A continent‐wide geospatial study on onchocerciasis highlighted the extent of underreported prevalence between 2000 and 2018 [21].

Surveys in KwaZulu‐Natal, South Africa, report high rates of schistosomiasis and soil‐transmitted helminths among rural schoolgirls [22]. In Togo, skin NTDs and fungal infections remain prevalent among peri‐urban and rural school‐aged children [23], reinforcing the need for targeted school‐based interventions.

Sudan bears a complex and substantial burden of endemic NTDs, including zoonotic variants. Armed conflict and fragile health infrastructure further complicate control efforts. A One Health approach has recently been advocated to integrate veterinary, environmental, and human health systems, especially in light of overlapping crises [24]. For instance, coinfections with dengue and malaria have been documented in febrile patients in Kassala, eastern Sudan, posing diagnostic and therapeutic challenges [25]. Human leishmaniasis remains widely distributed, with both cutaneous and visceral forms contributing to significant morbidity [26]. Blinding trachoma continues to afflict populations in northern states [27], and refugee populations in White Nile State exhibit high disease rates, highlighting the intersection between displacement and NTD risk [28].

Khartoum State, as Sudan’s capital and economic center, presents unique challenges due to urban density, widespread internal displacement, and fragile healthcare infrastructure [29]. Despite these known risk factors, there is a notable paucity of localized data on the spatial distribution and prevalence of NTDs in the state. Addressing this gap is essential for informed planning and effective intervention.

This study aimed to assess the prevalence and geographical distribution of NTDs in Khartoum State, Sudan, using annual statistical reports from the State Ministry of Health. The findings are intended to inform evidence‐based control strategies and support planning for the eventual elimination of these diseases.

## 2. Methods

### 2.1. Study Design

This study employed a retrospective cross‐sectional design to assess the prevalence, demographic distribution, and geographic clustering of NTDs in Khartoum State, Sudan. Secondary data were obtained from official public health surveillance records, providing a snapshot of NTD trends over a 2‐year period (2020‐2021).

### 2.2. Study Area

Khartoum State, the smallest of Sudan’s 18 states by area (22,142 km^2^), is the most populous, with 5,274,321 people recorded in the 2008 census and an estimated 9.4 million in 2023 (national population: 49.7 million). It includes Sudan’s largest city and national capital, Khartoum. Located at the confluence of the White and Blue Nile rivers, it spans longitudes 31.5° to 34°E and latitudes 15° to 16°N. The state comprises seven localities: Khartoum, Omdurman, Khartoum North, Sharq an‐Nīl, Jabal Awliya, Om Badda, and Karari (Figure 1). The area features a mix of urban, peri‐urban, and rural settings, with varied water sources and sanitation infrastructure, and hosts a significant presence of internally displaced persons (IDPs) and migrants [29].

**Figure FIGURE 1:**
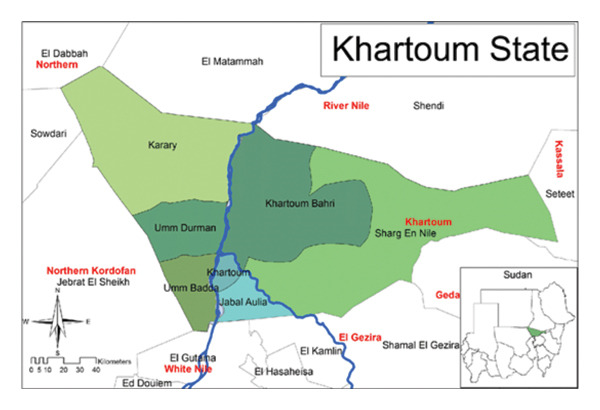
Map of Khartoum State showing the location and localities.

### 2.3. Data Sources

Annual statistical reports for 2020 and 2021 were obtained from the Khartoum State Ministry of Health. These reports compile surveillance data from public healthcare facilities across all localities. The datasets included confirmed cases of nine NTDs: soil‐transmitted helminthiases (STHs), schistosomiasis (*Schistosoma haematobium* and *Schistosoma mansoni*), scabies, trachoma, snakebite and scorpion envenoming, leishmaniasis (cutaneous and visceral), taeniasis, mycetoma, and leprosy. Demographic details (age and sex) and locality‐level distribution were included where available. Diagnostic confirmation was based on laboratory tests (e.g., stool and urine exams for parasitic infections and clinical examination for skin‐related NTDs).

### 2.4. Inclusion and Exclusion Criteria

#### 2.4.1. Inclusion Criteria

All reported and confirmed NTD cases were recorded in Khartoum State for 2020 and 2021.

Data entries with sufficient demographic and geographic identifiers (age, sex, and locality) were included.

#### 2.4.2. Exclusion Criteria

Incomplete or inconsistent records lacking diagnostic confirmation or location were included.

Duplicate entries.

### 2.5. Data Collection and Management

Data were extracted from PDF and tabular formats into Microsoft Excel for initial cleaning and organization. Data were checked for duplicates, missing values, and internal consistency (e.g., verifying age–sex–disease match). Age groups were categorized (< 1, 1–< 5, 5–< 15, 15–< 25, 25–< 45, 45–< 60, and ≥ 60 years) to facilitate stratified analysis.

### 2.6. Statistical Analysis

Descriptive statistics summarized disease prevalence, age and sex distribution, and annual trends using Microsoft Excel and SPSS Version 26. Analyses performed included the following:

Frequency distributions: Total and relative frequencies (%) of each NTD across years and localities.

Age and sex stratification: Cross‐tabulations of NTD types with age groups and sex to identify vulnerable populations.

Trend analysis: Year‐on‐year comparison to assess increasing, decreasing, or stable trends of NTD cases in each locality.

### 2.7. Spatial Analysis

Spatial distribution was generated for the three most prevalent NTDs: STH, *S. haematobium*, and *S. mansoni* (Tables 1, 2, and 3). Spatial clustering and changes in geographic distribution between 2020 and 2021 were evaluated to infer areas of persistent or emerging transmission.

**Table TABLE 1:** Distribution of soil‐transmitted helminthiases in Khartoum State by localities according to the stool test 2020‐2021.

Locality	2020		2021		Trend
	Cases	%	Cases	%	
Khartoum	6042	46.5	104	0.9	Decreasing
Jabal Awliya	2240	17.2	1463	12.5	Decreasing
Omdurman	762	5.9	367	3.2	Decreasing
Karary	935	7.2	764	6.5	Decreasing
Ombadda	479	3.7	2283	19.5	Increasing
Bahry	2152	16.5	486	4.1	Decreasing
Sharg Elniel	391	3.0	6247	53.3	Increasing
Total	13001	100	11714	100	Decreasing

**Table TABLE 2:** Distribution of *Schistosoma haematobium* in Khartoum State by localities according to the urine test 2020‐2021.

Locality	2020		2021		Trend
	Cases	%	Cases	%	
Khartoum	430	6.3	168	3.0	Decreasing
Jabal Awliya	4671	68.5	3891	68.7	Decreasing
Omdurman	123	1.8	163	2.9	Increasing
Karary	131	1.9	53	0.9	Decreasing
Ombadda	545	8.0	296	5.3	Decreasing
Bahry	233	3.5	384	6.8	Increasing
Sharg Elniel	683	10.0	705	12.4	Increasing
Total	6816	100	5660	100	Decreasing

**Table TABLE 3:** Distribution of *Schistosoma mansoni* in Khartoum State by localities according to the stool test 2020‐2021.

Locality	2020		2021		Trend
	Cases	%	Cases	%	
Khartoum	331	14.2	68	3.8	Decreasing
Jabal Awliya	818	35.2	444	24.8	Decreasing
Omdurman	201	8.7	164	9.2	Decreasing
Karary	128	5.5	95	5.3	Decreasing
Ombadda	309	13.3	409	22.8	Increasing
Bahry	245	10.5	225	12.6	Decreasing
Sharg Elniel	294	12.6	385	21.5	Increasing
Total	2326	100	1790	100	Decreasing

### 2.8. Ethical Considerations

This study was based solely on anonymized secondary data obtained from the Ministry of Health and did not involve direct contact with patients or collection of personal identifiers. Permission to access and analyze the data was granted through an official letter from the Director General of Khartoum State Ministry of Health.

## 3. Results

A total of nine out of the 21 NTDs identified by the World Health Organization (WHO) were found to be prevalent in Khartoum State. These included STHs, schistosomiasis, scabies, trachoma, snakebite envenoming, leishmaniasis, taeniasis, mycetoma, and leprosy (Figure 2).

**Figure FIGURE 2:**
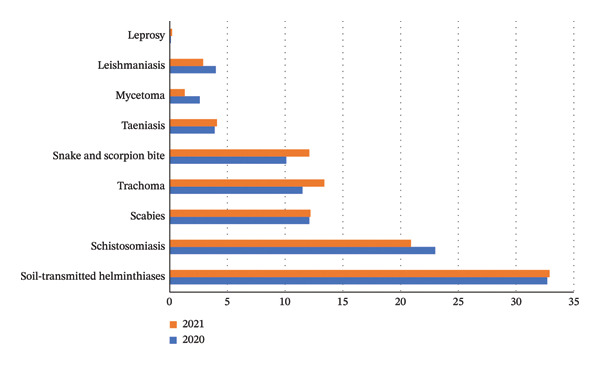
Percentage of prevalence of NTDs in Khartoum State in years 2020 and 2021.

### 3.1. STHs

STH was the most prevalent NTD, accounting for 32.7% of total NTD cases in 2020 and 32.9% in 2021. The distribution between sexes was nearly equal, with males representing 49.3% and females 50.7% of the cases. In 2020, STH was most prevalent in the localities of Khartoum (46.5%), Jabal Awliya (17.2%), and Bahry (16.5%). In 2021, the highest prevalence was recorded in Sharg Elniel (53.3%), followed by Ombadda (19.5%) and Jabal Awliya (12.5%) (Figure 3). More than half (51%) of STH infections occurred in individuals under 15 years of age.

**Figure FIGURE 3:**
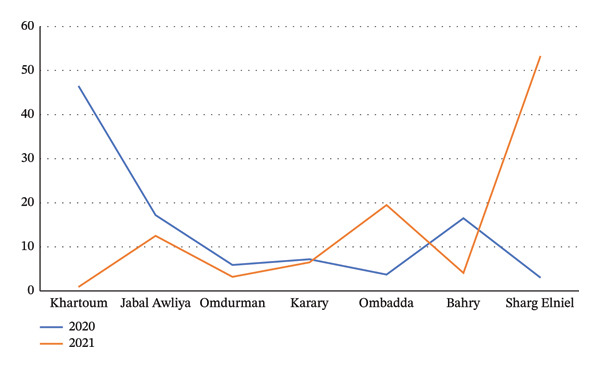
Percentage of distribution of soil‐transmitted helminthiases in Khartoum State localities according to stool tests from 2020 to 2021.

### 3.2. Schistosomiasis

Schistosomiasis was the second most prevalent NTD, comprising 23.0% and 20.9% of NTD cases in 2020 and 2021, respectively. The disease was significantly more prevalent among males (72.4%). In 2020, *S. haematobium* constituted 74.6% of schistosomiasis cases, while *S. mansoni* accounted for 25.4%. In 2021, the proportions were 68.4% for *S. haematobium* and 31.6% for *S*. *mansoni*.

In 2020, *S. haematobium* was most prevalent in the localities of Jabal Awliya (68.5% of cases), Sharg Elniel (10.0%), and Ombadda (8.0%) (Figure 4). These distributions may be associated with the presence of irrigation schemes in these areas. In 2021, the highest prevalence of *S. haematobium* remained in Jabal Awliya (68.7%), followed by Sharg Elniel (12.4%).

**Figure FIGURE 4:**
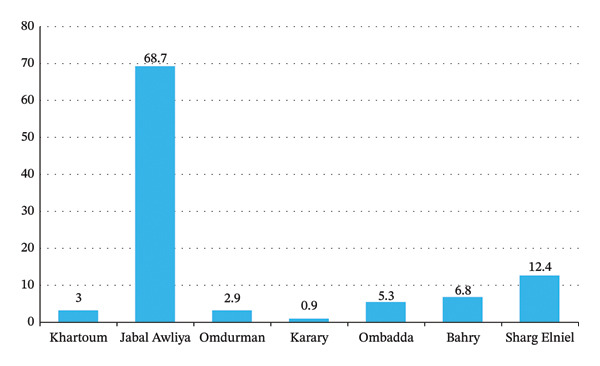
Percentage of the distribution of *Schistosoma haematobium* in Khartoum State localities according to urine tests in 2021.

*S. mansoni* was most prevalent in 2020 in Jabal Awliya (35.2%), Khartoum (14.2%), and Sharg Elniel (12.0%) (Figure 5). In 2021, it continued to be concentrated in Jabal Awliya (24.8%), Ombadda (22.8%), and Sharg Elniel (21.5%). Across both years, 52.6% of schistosomiasis cases were among individuals aged 5–25 years.

**Figure FIGURE 5:**
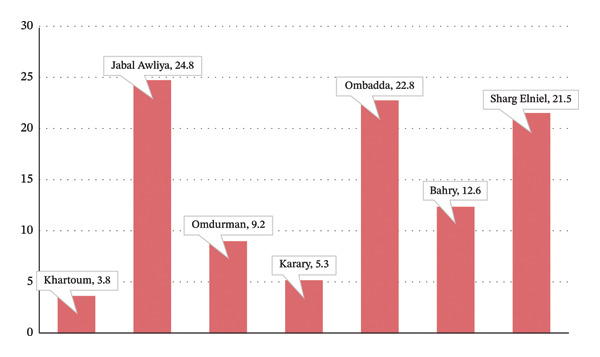
Percentage of distribution of *Schistosoma mansoni* in Khartoum State localities according to stool tests in 2021.

### 3.3. Scabies

Scabies affected individuals across all age groups, from under 1 year to over 60 years of age. Males were more affected than females, comprising 60.6% of cases in 2020 and 58.7% in 2021. The disease was most prevalent among individuals under 15 years old (Table 4).

**Table TABLE 4:** Distribution of five NTDs in Khartoum State by age and sex, 2020‐2021.

Age	Sex	Disease									
		Sc.		SSB		Myc		Leish		Lep.	
		2020	2021	2020	2021	2020	2021	2020	2021	2020	2021
< 1 year	M	154	91	10	7	19	17	35	9	0	0
	F	135	74	12	12	20	13	63	7	0	1

1–< 5	M	403	369	133	159	33	45	100	37	0	2
	F	241	236	150	181	48	48	121	34	0	0

5–< 15	M	686	540	344	382	50	52	199	85	2	3
	F	302	287	311	323	35	59	118	71	0	0

15–< 25	M	514	445	432	455	34	35	151	119	3	2
	F	341	313	476	541	137	41	82	87	1	5

25–< 45	M	624	557	563	572	58	42	221	154	8	16
	F	502	483	677	698	72	48	105	129	5	14

45–< 60	M	298	321	36	404	150	14	174	122	7	14
	F	222	235	247	294	168	30	95	111	2	6

60–> 60	M	222	223	152	159	75	11	75	71	4	9
	F	146	165	142	112	117	4	64	15	2	9

Total	M	2901	2546	1999	2138	419	216	955	596	24	46
	F	1889	1793	2015	2161	597	243	648	454	10	35

Grand total		4790	4339	4014	4299	1016	459	1603	1050	34	81

% From gr. Total	M	60.6	58.7	49.8	49.7	41.2	47.1	59.6	56.8	70.6	56.8
	F	39.4	41.3	50.2	50.3	58.8	52.9	40.4	43.2	29.4	43.2

*Note:* M: male, F: female, Sc.: scabies, Myc.: mycetoma, Liesh.: leishmaniasis, and Lep.: leprosy.

Abbreviation: SSB, snake and scorpion bite.

### 3.4. Trachoma

In 2020, trachoma was most prevalent in Khartoum (31.6%), Omdurman (31.4%), Jabal Awliya (18.9%), and Karary (17.3%). In 2021, the highest prevalence was reported in Omdurman (47.5%), followed by Khartoum (25.3%), Jabal Awliya (13.9%), and Karary (12.8%). Sharg Elniel was nearly free of trachoma, with only one recorded case in 2020 (Table 5).

**Table TABLE 5:** Distribution of trachoma in Khartoum State by localities according to stool tests from 2020 to 2021.

Locality	2020		2021		Trend
	Cases	%	Cases	%	
Khartoum	1449	31.61	1216	25.3	Decreasing
Jabal Awliya	865	18.87	665	13.9	Decreasing
Omdurman	1437	31.35	2277	47.5	Increasing
Karary	792	17.28	612	12.8	Decreasing
Ombadda	2	0.04	17	0.4	Increasing
Bahry	38	0.83	7	0.1	Decreasing
Sharg Elniel	1	0.02	0	0	Decreasing
Total	4584	100	4794	100	Increasing

### 3.5. Snakebite and Scorpion Envenoming

Snakebite and scorpion envenoming, reported together, had a combined prevalence of 10.2% (4014/39,373) of all NTD cases in 2020 and 11.4% (4299/37,690) in 2021. These cases were not disaggregated in the source reports; however, the combined data indicate a significant burden. Affected individuals spanned all age groups, with a similar prevalence between males and females. The most affected age group was 25 to < 45 years (Table 4).

### 3.6. Leishmaniasis

Leishmaniasis was reported in all age groups, with the highest prevalence among individuals aged 15 to < 45 years. The disease affected males more than females, with male cases representing 59.6% in 2020 and 56.8% in 2021. Cutaneous leishmaniasis constituted 97.4% of all cases, while visceral leishmaniasis made up only 2.6% (Table 4).

### 3.7. Taeniasis

Taeniasis showed an almost equal sex distribution, with a slight increase among females. The most affected age group was 15 to < 45 years. In 2020, the disease was most prevalent in Jabal Awliya (28.5%) and Ombadda (23.1%). In 2021, the highest prevalence was recorded in Ombadda (56.1%), followed by Jabal Awliya (21.8%) and Omdurman (11.1%) (Table 6).

**Table TABLE 6:** Distribution of taeniasis in Khartoum State by localities according to stool tests from 2020 to 2021.

Locality	2020		2021		Trend
	Cases	%	Cases	%	
Khartoum	176	11.5	15	1.0	Decreasing
Jabal Awliya	437	28.5	173	11.8	Decreasing
Omdurman	84	5.5	163	11.1	Increasing
Karary	128	8.3	47	3.2	Decreasing
Ombadda	354	23.1	822	56.1	Increasing
Bahry	138	9.0	41	2.8	Decreasing
Sharg Elniel	217	14.1	205	14.0	Decreasing
Total	1534	100	1466	100	Decreasing

### 3.8. Mycetoma

Mycetoma affected all age groups and was more common among females, who comprised 58.8% of cases in 2020 and 52.9% in 2021 (Table 4).

### 3.9. Leprosy

Leprosy was the least prevalent NTD in Khartoum State, constituting only 0.2% of total NTD cases across 2 years. However, detection showed an increasing trend (Table 4).

## 4. Discussion

This study highlights the substantial burden of NTDs in Khartoum State, where nine of the 21 NTDs identified by the WHO were reported during the study period. These include STHs, schistosomiasis, scabies, trachoma, snakebite envenoming, leishmaniasis, taeniasis, mycetoma, and leprosy. The analysis provides important insights into epidemiological trends and demographic distributions, with implications for targeted public health strategies.

STH emerged as the most prevalent NTD in both 2020 and 2021, constituting approximately one‐third of all reported NTD cases (32.7% in 2020 and 32.9% in 2021). The near‐equal sex distribution suggests that environmental exposure, rather than behavioral differences, is the primary transmission pathway. Notably, over half of STH cases (51%) were reported in individuals under 15 years of age, identifying school‐aged children as a particularly vulnerable group. The shift in geographic hotspots—from Khartoum, Jabal Awliya, and Bahry in 2020 to Sharg Elniel and Ombadda in 2021—may reflect spatial variations in sanitation, water contamination, and population mobility.

Schistosomiasis was the second most common NTD, with a modest decline in prevalence from 23.0% in 2020 to 20.9% in 2021. The predominance among males (72.4%) likely reflects occupational exposure, especially in contexts involving contact with contaminated water. *S. haematobium* was the dominant species, particularly in Jabal Awliya—an area characterized by irrigation schemes and proximity to water bodies conducive to the parasite’s lifecycle. The most affected age group (5–25 years) overlaps with school attendance and early labor force participation, underscoring the value of school‐based health interventions.

Scabies affected individuals across all age groups but was particularly prevalent among males and children under 15. Its transmission dynamics—linked to overcrowding, close physical contact, and poor hygiene—align with common conditions in school and household settings. The gender disparity warrants further investigation into exposure and behavioral risk factors.

Trachoma exhibited a slight overall increase from 2020 to 2021, with a notable rise in cases in Omdurman, contrasting with declining trends in other localities. This localized resurgence suggests the persistence of transmission pockets and highlights the need for intensified hygiene promotion and environmental sanitation efforts.

Snakebite envenoming showed uniform distribution across sexes and age groups, with peak incidence in adults aged 25 to < 45 years. This likely reflects occupational exposure during agricultural and outdoor activities. These findings emphasize the need for broader access to antivenom and community‐level training in prevention and first aid.

Leishmaniasis, predominantly cutaneous (97.4%), was most prevalent among males aged 15 to < 45, a group typically engaged in outdoor labor. The psychosocial and economic consequences of cutaneous leishmaniasis, including disfigurement and stigma, underscore the importance of early detection and vector control.

Taeniasis maintained stable overall prevalence, although there were notable changes in geographic distribution, with Ombadda surpassing Jabal Awliya in 2021. This shift may be due to differences in meat inspection practices, sanitation, and food safety standards. The slightly higher prevalence among females may be associated with the roles in food preparation and potential exposure to contaminated meat.

Mycetoma showed a higher prevalence among females across a wide age range. This pattern could reflect increased environmental exposure or delayed care‐seeking behavior among women. Given its chronic progression and potential for disability, early detection and access to specialized treatment services are critical.

Leprosy accounted for only 0.2% of total NTD cases but showed a rising trend, which is concerning given its capacity to cause severe disability and stigma. Its presence across both sexes and all age groups, with a male predominance, suggests ongoing community transmission and reinforces the need for strengthened surveillance and proactive case‐finding.

A comparative analysis with the existing literature supports the relevance of these findings. Pone et al. [17], in their study in Dschang, West Cameroon, reported an overall infection prevalence of 43.5%, with STH as the most common infection (32.7% in 2020 and 32.9% in 2021), consistent with our results. However, unlike Pone et al., who focused on general infection rates, our study provides a more nuanced view, incorporating trend data, geographic variation, and sex‐specific differences. While they reported higher infection rates among females and a peak in the 21–30 age group, we observed greater prevalence among males and children under 15. Additionally, we noted declining trends in most NTDs, excluding trachoma, snakebite envenoming, and leprosy, which were not examined for temporal variation in Pone et al.’s work.

Similarly, Zulu et al. [22] reported a 32.2% prevalence of *S. haematobium* among school‐aged children in KwaZulu‐Natal, South Africa. While our study reported lower prevalence (23.0% in 2020 and 20.9% in 2021), the concentration among younger age groups is consistent. Unlike Zulu et al., who focused on helminth intensity, our study offers a broader epidemiological landscape encompassing multiple NTDs and their demographic and spatial distributions.

### 4.1. Public Health Implications

• Targeted interventions are essential for school‐aged children and young adults, particularly for STH and schistosomiasis control.
• Enhanced investment in water, sanitation, and hygiene (WASH) infrastructure is vital to interrupt transmission of helminths and trachoma.
• Vector control and environmental management strategies should be prioritized to address schistosomiasis, leishmaniasis, and mycetoma.
• Community‐based education on snakebite prevention and scabies management can reduce morbidity and improve treatment outcomes. For snakebites, enhanced surveillance with case disaggregation is needed to guide antivenom supply chains.
• Strengthening surveillance systems and early detection mechanisms is critical for managing low‐prevalence, high‐impact diseases such as leprosy.

### 4.2. Limitations

The study is limited to Khartoum State and may not reflect the NTD burden in other regions of Sudan. Additionally, reliance on routine surveillance data introduces potential reporting biases and variability in data quality. Specifically, the aggregation of snakebite and scorpion sting cases limits the operational utility of the data for planning specific antivenom procurement and distribution, a key consideration for national control strategies.

## 5. Conclusion

This study reveals a significant and heterogeneous burden of NTDs in Khartoum State during 2020‐2021, with STH and schistosomiasis emerging as the most prevalent, particularly among school‐aged children and young adults. The observed geographic and demographic disparities in NTD distribution reflect the complex interplay of environmental factors, population mobility, and inequitable access to water, sanitation, and healthcare services. Although some NTDs demonstrated declining trends, the rising incidence of trachoma, snakebite envenoming, and leprosy indicates evolving public health concerns that warrant immediate attention. The persistence of NTDs across all demographic groups underscores the urgent need for integrated, context‐specific interventions. Strengthening disease surveillance, enhancing WASH infrastructure, expanding access to diagnostics and treatment, and promoting community‐based health education should be prioritized within a comprehensive strategy to control and ultimately eliminate NTDs in the region.

## Funding

No funding was received for this manuscript.

## Conflicts of Interest

The authors declare no conflicts of interest.

## Data Availability

The data that support the findings of this study are available on request from the corresponding author. The data are not publicly available due to privacy or ethical restrictions.
